# Knowledge and Perceptions of College Students Regarding the Physician Assistant Profession

**DOI:** 10.7759/cureus.368

**Published:** 2015-10-28

**Authors:** Mark Volpe, Sandra Bulmer, Chandra Kelsey

**Affiliations:** 1 School of Medicine, Yale University; 2 School of Health and Human Services, Southern Connecticut State University; 3 Department of Public Health, Southern Connecticut State University

**Keywords:** physician assistant, medical education, college students, knowledge, perceptions

## Abstract

*Purpose*: Physician assistants (PAs) are nationally certified and state-licensed medical professionals who practice medicine on healthcare teams with physicians and other providers. Despite the increasing popularity and utility of the profession, knowledge of the role of PAs remains scarce among many segments of the population. The purpose of this study was to determine the knowledge and perceptions of the PA profession among undergraduate college students, as well as what factors are associated with better knowledge and perception of the profession.

*Methods*: Using a cross-sectional survey, information was gathered regarding knowledge and perception of PAs. A total of 364 students were surveyed from randomly selected undergraduate courses at a Connecticut public university.

*Results*: Knowledge scores were significantly higher (p<0.05) in older students, female students, those with plans to pursue a healthcare career, those majoring in health and human services, and those satisfied with care received from a PA. Significantly better perceptions (p<0.05) of PAs were found in older students, those with plans for a future career in healthcare, those majoring in health and human services, those who received prior care from a PA, and those who were satisfied with prior care from a PA. After a short educational intervention, improvements in perceptions were statistically significant (p<0.001) in the surveyed population.

*Conclusion*: This study identifies areas of needed improvement in knowledge and perception of PAs and also provides impetus for educational and marketing-based interventions to improve knowledge and perception of the PA profession in the college student population.

## Introduction

Physician assistants (PAs) are nationally certified and state-licensed medical professionals who practice medicine on healthcare teams with physicians and other providers [1]. Despite the increasing popularity and utility of the physician assistant (PA) profession, recent peer-reviewed studies evaluating the public knowledge and perception of PAs remains scarce. There are several studies in the emergency medicine patient population that found generally positive perceptions of PAs [2-4]. However, there is still room for improvement as one study of the emergency medicine patient population found that almost 40% of patients would not be willing to be cared for by a PA, regardless of their injury [5]. Moreover, several studies of the perception of PAs among the primary care population have found that patients are generally satisfied with PA care and that patients would rather see a PA or nurse practitioner than wait to see a physician [6-8]. However, in the veteran population, patient satisfaction scores did not improve when more PAs were added to care teams but did improve when more physicians or nurse practitioners were added to care teams [9]. Clearly, further research is needed to better elucidate the perception of PAs among these and other populations.

Additionally, the only national survey to examine knowledge of the role of the PA within the United States found that though 66% of respondents had heard of the PA profession, only approximately 20% were able to accurately describe what a PA actually does [10]. This presents an important public health issue for several reasons. First and foremost, the public deserves to know what type of provider is caring for them, as well as the skills, expertise, and knowledge of that provider so they can make informed decisions regarding their healthcare. Additionally, as the number of PAs continues to increase, more patients will be provided medical care by PAs and should thus be knowledgeable about the profession. Also, in providing cost-effective health care, PAs will likely see increased roles throughout medicine in attempts to curb costs. Furthermore, public knowledge of the profession is critical in attracting the brightest minds into the field and ensuring future continued growth and development of the PA profession.

The purpose of this study is to determine the knowledge and perceptions of the PA profession among undergraduate college students, as well as what factors are associated with better knowledge and perception of the profession. This population was chosen as it represents a population that (1) has not yet been studied; (2) will likely receive some of their future health care from PAs; (3) may influence scope of practice and care in the future; and (4) may themselves become healthcare professionals that interact with PAs on a daily basis. Determining the knowledge and perceptions of this population is critical because this information will enable the PA workforce and PA students to focus their efforts on narrowing knowledge gaps and improving perceptions. Furthermore, it is relevant to PA educators and policy makers who can utilize these results to better create educational and marketing materials targeted towards college students receiving care from a PA and target gaps in knowledge and poor perceptions among those considering pursuing a career as a PA in the future. Finally, these results can be utilized to tailor health promotion and professional advocacy components of PA curricula to provide PA students with the skills and tools needed to improve knowledge and perception of the profession as new graduate PAs enter the workforce.

## Materials and methods

This study used a cross-sectional design and descriptive survey to examine the knowledge and perceptions of college students regarding the PA profession. The independent variables measured by the descriptive survey included age, race, gender, subject of study, class standing, plans to pursue a health-related profession, class standing, receipt of care from a PA, and satisfaction with care from a PA. The dependent variables included knowledge and perceptions.

### Study population

The study population included undergraduate students at a public university in Connecticut housing Bachelor’s, Master’s, and Doctorate degree programs in a wide array of arts, sciences, healthcare, human services, education, and business related fields. At the time of this study, the undergraduate population at this university was comprised of 71.5% Caucasian undergraduates and 28.5% minorities (most of which were African-American or Hispanic) and approximately 7,000 full-time undergraduate students, with nearly 70.0% females and 30.0% males [11].

To be included in this study, students must have been full-time undergraduate students, aged 18-23. This study excluded part-time students, students studying abroad, and undergraduate students under 18 and over 23 as these students do not represent the typical undergraduate that this study aims to characterize.

Participants who agreed to participate were explained the nature and the objectives of the study, and informed consent was formally obtained.

### Sampling procedures

A simple random sample of classes was utilized to select participants to complete the survey. Professors of each randomly selected course were contacted via email inquiry to ask to participate in the study. Of the 16 classes chosen and professors emailed, 10 professors responded positively and allowed the researchers to survey their class. Overall, 474 students were sampled. The response rate was 86.7%. A total of 364 returned surveys were required to achieve 80% power to detect a medium effect size at the α = .05 significance level among the 7,014 full-time undergraduate students. A total of 411 surveys were completed from February 11, 2015 through February 20, 2015. A total of 47 students were ineligible for the study because they were greater than 23 years old, leaving 364 eligible participants. There were no students who were ineligible for any of the other exclusion criteria. Importantly, students were instructed before each survey administration that if they have already completed the survey in a different class then they should not fill out the survey again.

### Institutional review board

Two institutional review board (IRB) submissions were required to complete this study. One IRB submission was completed for pilot testing and cognitive interviewing of pilot testers of the survey instrument. The second IRB submission was submitted to conduct the actual study. Both IRB submissions were approved by Southern Connecticut State University Institutional Review Board (approval #14-191 and #14-192) where the study took place.

### Instrumentation

A survey instrument was used to collect data regarding the knowledge and perception of the PA profession among undergraduate students. The instrument contained demographic questions regarding age, gender, race, year of education, school enrollment, and plans to pursue a health-related career path in the future. All knowledge and perception questions were multiple choice or yes/no format. Additionally, participants were given a description of the PA profession (short educational intervention) at the end of the survey with subsequent questions to determine if new knowledge altered their perception of the profession. This intervention consisted of a one-page document that included a description of the PA profession, the educational requirements, and typical duties and responsibilities.

To ensure reliability and validity of the instrument, several steps were taken. To assess reliability, internal consistency was determined by calculating an alpha coefficient for items measuring the same general construct. The alpha coefficient for the knowledge questions was 0.902, indicating excellent reliability in measuring the knowledge construct. The alpha coefficient for the perception questions was 0.924, indicating excellent reliability in measuring the perception construct. To assess validity, two steps were taken. Content validity was assessed through several cycles of expert review by two practicing PA educators and one public health professor with extensive survey development experience. The instrument was approved by all of these individuals as measuring intended constructs, given the goal of the research project, before it was administered. Additionally, a cognitive interview pilot study was also conducted (described below).

### Pilot test

A pilot test was conducted as part of the validation project after IRB approval. The testing consisted of cognitive interviews with five undergraduate students to receive feedback regarding the survey. Participants were recruited from public health and social work clubs on campus via emailing student group leaders. Students completed the preliminary survey instrument. Next, they were asked to provide feedback regarding directions, content, cultural sensitivity, item appropriateness, format, materials needed to complete the instrument, readability, item clarity, and ease of administration. Any problems encountered or feedback given regarding the survey instrument was utilized to produce a final survey instrument that was administered as part of the study after final approval from the expert panel.

### Data collection procedures

Data were collected from students in randomly selected classes as previously described. The primary investigator or a research assistant began by reading a brief introductory statement regarding the survey purpose and mentioned that the survey was completely voluntary. The survey instrument was administered in paper form to those who chose to participate. After it was completed, students placed the survey into a folder anonymously. The completed surveys were then entered into the Statistical Package for the Social Sciences (SPSS) version 20 and analyzed by the primary investigators.

### Data analysis procedures

All analyses in this study were completed using the SPSS software. Baseline demographic, knowledge, and perception data were compiled using descriptive summaries and frequency distributions. Furthermore, the association between demographic variables and knowledge, demographic variables and perception, as well as perception before and after being provided with the short educational intervention was tested. To compare demographic variables and knowledge as well as demographic variables and perception, student t-tests or ANOVA at *p* = .05 were performed, based on the number of categories tested. To compare perceptions of the PA profession before and after being provided with the short educational intervention, a paired t-test at *p* = .05 was conducted. Finally, to compare responses to perception questions before and after the intervention, chi-square tests at *p *= .05 were performed. As previously mentioned, an alpha coefficient was calculated to test internal consistency reliability of the instrument.

Knowledge and perception scores were calculated as follows. Each correct knowledge question was given two points so that a total knowledge score was calculated for each completed survey. Incorrect knowledge responses were given zero points. Unanswered knowledge questions or questions answered as “I don’t know” were given one point. The range of possible knowledge scores was 0 to 68. Each perception question answered with a positive response was given two points, and those answered with a negative perception response given zero points, and unanswered perception questions or questions answered neutrally were given one point. The range of possible perception scores was 0 to 34. A change in perception score was calculated by subtracting each student’s post-perception score from their baseline perception score.

## Results

### Baseline characteristics

Table 1 presents the baseline characteristics of the surveyed college population. Of note, the majority of participants were 18 to 19 years old, female, white, and non-Hispanic. Approximately half of students participating replied that they plan to pursue a future career in healthcare, and about half were majoring in a health or human services field. Most of the respondents reported receiving care from a PA and being satisfied with that care.

**Table 1:** Baseline Characteristics of Surveyed Undergraduate Students (n=364) Percentages are of total responses to a given question. Frequencies do not add up to 364 as not all respondents answered each question.

Characteristic	*f*	Percent
Age		
18	104	28.8
19	102	28.3
20	71	19.7
21	44	12.2
22	27	7.4
23	13	3.6
Gender		
Male	82	22.7
Female	275	76.2
Deferred	4	1.1
Race		
White	249	69.9
Black or African-American	56	15.7
Native Hawaiian or Pacific Islander	0	0.0
Asian	12	3.4
American Indian or Alaskan Native	1	0.3
Other	32	9.0
Deferred	6	1.7
Ethnicity		
Hispanic	44	12.7
Non-Hispanic	292	84.1
Deferred	11	3.2
Future career in healthcare		
Yes	168	46.7
No	165	45.8
Undecided	27	7.5
Current major		
Arts and sciences	86	24.4
Health and human services	185	52.6
Business	30	8.5
Education	51	14.5
Year in school		
Freshman	147	41.0
Sophomore	105	29.2
Junior	57	15.9
Senior	50	13.9
Received care from PA		
Yes	208	57.9
No	50	13.9
Unsure	101	28.2
Satisfaction with care from PA		
Yes	191	58.8
No	9	2.8
Unsure	125	38.4

### Knowledge results

The mean knowledge scores subdivided by baseline characteristics are provided in Table 2. There were several statistically significant associations discovered and trends. As students increased in age, their average knowledge score also increased. The average knowledge score of 18 to 19-year-olds was 35.17 while that of 22 to 23-year-olds was 40.90 (*p*=0.002). Females also had significantly more knowledge regarding the PA profession than males (*p*=0.041). As expected, students who had planned a future career in health care had significantly higher knowledge scores than those who did not (*p*< 0.001), as did students majoring in health or human services disciplines compared to those with majors in education (*p*=0.002). Furthermore, upperclassmen showed more knowledge of the profession than underclassmen (*p*<0.001), and those who were satisfied with their previous care from a PA had more knowledge than those who were unsatisfied (*p*=.004).

There were no significant associations found when comparing knowledge scores by race or ethnicity. Additionally, knowledge scores did not significantly differ based on whether or not participants were previously cared for by PAs. Comparisons of knowledge scores among the other major fields of study were not statistically significant.

**Table 2:** Knowledge Score of Surveyed Undergraduate Students by Baseline Characteristics * Comparison statistically significant at *p*<0.05. All *p*-values <.05 represent comparisons between the highest and lowest knowledge score for the given characteristic. All *p*-values > .05 represent initial ANOVA or student t-test analyses and no post-hoc testing was performed given *p*-value >.05. ! Comparison between 18-19 and 20-21 year olds also significant at *p*=.011 @ Comparison between Education and Health and Human Services majors. Other comparisons were not significant.

Characteristics	Knowledge Score (Mean)	Standard Deviation	*p*-value
Age			
18-19	35.17	9.07	0.002*^!^
20-21	38.49	9.65	
22-23	40.86	9.10	
Gender			
Male	34.81	8.81	0.041*
Female	37.35	9.58	
Race			
White	37.33	9.64	0.193
Black or African-American	34.67	9.77	
Other	36.24	7.95	
Ethnicity			
Hispanic	36.90	7.91	0.999
Non-Hispanic	36.90	9.56	
Future career in healthcare			
Yes	39.25	10.18	<0.001*
No / undecided	34.63	8.22	
Major			
Arts and sciences	36.15	9.44	0.002*^@^
Health and human services	38.29	9.68	
Business	36.21	7.78	
Education	32.58	8.63	
Year of education			
Underclassmen (FR/SO)	35.28	8.87	<0.001*
Upperclassmen (JR/SR)	40.54	9.88	
Received care from PA			
Yes	37.59	10.05	0.086
No / unsure	35.77	8.49	
Satisfied with care from PA			
Yes	38.24	9.82	0.004*
No / unsure	35.05	9.52	

Table 3 provides more specific results on areas of knowledge that were measured with this survey. Among the knowledge questions, participants were asked whether or not they thought that PAs were able to perform certain medical tasks. Of note, 93.8% of participants thought that PAs were able to take vital signs, 90% thought that PAs could measure height and weight, 82.1% thought PAs could take a medical history, and 77.7% realized that PAs were not able to perform surgery. In contrast, less than 50% of participants thought that PAs could administer medications, interpret lab tests or x-rays, treat patients in the emergency room, or treat patients with chronic medical problems. Furthermore, less than 30% of participants thought that PAs could prescribe medications or be primary care providers. Notably, many participants were unsure whether or not PAs could perform certain tasks, ranging from 4.8% to 26.5% of responses, depending on the task assessed.

**Table 3:** Knowledge of PA Ability to Perform Tasks

Task	Yes *f* (%)	No *f* (%)	Unsure *f* (%)
Take vital signs	332 (93.8)	5 (1.4)	17 (4.8)
Give injections	255 (72.0)	66 (18.6)	33 (9.3)
Draw blood	264 (75)	57 (16.2)	31 (8.8)
Administer meds	155 (44)	162 (46)	35 (9.9)
Take medical history	289 (82.1)	34 (9.7)	29 (8.2)
Staple closed a head wound	111 (31.4)	172 (48.6)	71 (20.1)
Perform physical exam	223 (63.7)	89 (25.4)	38 (10.9)
Order lab tests	197 (56.0)	100 (28.4)	55 (15.6)
Order x-rays	187 (52.4)	108 (30.3)	57 (16.0)
Interpret lab tests	160 (45.7)	129 (36.9)	61 (17.1)
Interpret x-rays	161 (45.7)	131 (37.2)	60 (17.0)
Diagnose medical problems	123 (34)	174 (49.3)	56 (15.9)
Prescribe meds	103 (29.3)	209 (59.4)	40 (11.4)
Perform surgery	37 (10.6)	272 (77.7)	41 (11.7)
Treat minor medical problems	276 (78.6)	41 (11.7)	34 (9.7)
Treat chronic medical problems	123 (35.0)	161 (45.9)	67 (19.1)
Perform a skin biopsy	74 (21.1)	184 (52.4)	93 (26.5)
Counsel patients on smoking cessation	245 (70.0)	45 (12.9)	60 (17.1)
Educate patients	295 (83.8)	26 (7.4)	31 (8.8)
Refer patients to specialists	240 (68.4)	66 (18.8)	45 (12.8)
Treat patients in the emergency room	138 (39.2)	133 (37.8)	81 (23.0)
Be on call overnight	217 (61.5)	64 (18.1)	72 (20.4)
Be the primary care provider	81 (22.9)	209 (59.2)	63 (17.8)
Suture closed a patient after an operation	108 (30.5)	154 (43.5)	92 (26.0)
Apply casts	211 (59.8)	76 (21.5)	66 (18.7)
Deliver babies	78 (22.2)	204 (58.0)	70 (19.9)
Perform CPR	270 (76.7)	44 (12.5)	38 (10.8)
Measure height and weight	320 (90.9)	12 (3.4)	20 (5.7)
Practice independently	74 (20.9)	207 (58.5)	73 (20.6)

### Perception results

The mean perception scores subdivided by baseline characteristics are provided in Table 4. There were several significant associations discovered and trends. As students aged, the trend was to adopt a better perception of PAs with average scores of 19.81 from age 18-19 to 24.75 for students aged 22-23 (*p*=0.002). Those with plans for a future career in healthcare had a significantly better perception of PAs than those without plans to pursue a career in healthcare (*p*<0.001) as did upperclassmen as compared to underclassmen (*p*<0.001). Finally, those who were satisfied with care from a PA had significantly higher scores than those who were unsatisfied with their care (*p*=0.005). There were no significant associations found when examining perception scores by race, ethnicity, major, and gender.

**Table 4:** Initial Perception Score of Surveyed Undergraduate Students by Baseline Characteristics * Comparison is statistically significant at *p*<0.05. All *p*-values <0.05 represent comparisons between the highest and lowest knowledge score for the given characteristic All *p*-values > .05 represent initial ANOVA analyses and no post-hoc testing was performed given *p*-value >.05. ! Comparison between 18-19 and 22-23 year olds is significant at *p*=0.002. Comparison between 18-19 and 20-21 year olds is significant at *p*= 0.025.

Characteristics	Perception Score (mean)	*p*-value
Age		
18-19	19.81	0.002*^!^
20-21	22.14	
22-23	24.75	
Gender		
Male	20.70	0.254
Female	21.33	
Race		
White	20.70	0.230
Black or African-American	21.33	
Other	20.70	
Ethnicity		
Hispanic	21.55	0.784
Non-Hispanic	21.16	
Future career in healthcare		
Yes	23.08	<0.001*
No / undecided	19.27	
Major		
Arts and sciences	19.87	0.066
Health and human services	22.28	
Business	19.75	
Education	19.69	
Year of education		
Underclassmen (FR/SO)	19.82	<0.001*
Upperclassmen (JR/SR)	24.06	
Received care from PA		
Yes	21.87	0.039*
No / unsure	19.94	
Satisfied with care from PA		
Yes	22.43	0.005*
No / unsure	19.69	

In comparing the initial and final perception scores pre- and post-intervention among all participants, a statistically significant increase in score was noted at *p*<0.001. The mean initial score was 21.69 while the mean final score was 26.80. Initial and final perception scores between each subgroup varied from a low initial score of 19.07 in African Americans to a high final score of 29.41 for the 22 to 23-year-old age group. The mean change in score among the subgroups varied from 3.71 in upperclassmen to 7.11 in those who were unsatisfied with care from a PA (Table 5).

**Table 5:** Comparison of Initial and Final Perception Score of Surveyed Undergraduate Students by Baseline Characteristics Comparison between average initial and final perception score (21.69 and 26.80 respectively) is statistically significant at *p*<0.001.

Characteristics	Initial Perception Score	Final Perception Score	Mean Change in Score
Age			
18-19	19.81	26.43	6.62
20-21	22.14	26.37	4.23
22-23	24.75	29.41	4.66
Gender			
Male	20.70	24.64	3.96
Female	21.33	27.32	6.01
Race			
White	20.70	26.76	5.44
Black or African-American	21.33	25.58	6.51
Other	20.70	26.95	5.59
Ethnicity			
Hispanic	21.55	28.89	7.34
Non-Hispanic	21.16	26.74	5.58
Future career in healthcare			
Yes	23.08	28.71	5.63
No / Undecided	19.27	24.99	5.72
Major			
Arts and sciences	19.87	25.69	5.82
Health and human services	22.28	27.92	5.64
Business	19.75	25.13	5.38
Education	19.69	25.48	5.79
Year of education			
Underclassmen (FR/SO)	19.82	26.35	6.53
Upperclassmen (JR/SR)	24.06	27.77	3.71
Received care from PA			
Yes	21.87	26.77	4.90
No / unsure	19.94	26.79	6.85
Satisfied with care from PA			
Yes	22.43	26.93	4.50
No / unsure	19.69	27.80	7.11

Table 6 provides a summary of analyses that were conducted to examine the initial and final perception of students regarding the ability of PAs to provide care for different medical problems. In each case, a significant improvement in perception was noted (*p*<0.05), with the exception of “cold or flu symptoms” which already had a very high percentage indicating a positive response at baseline. Initial perception responses among respondents indicating confidence in PA care ranged from 21.6% for major car accident to 88.9% for cold or flu symptoms. Final perception responses indicating confidence in PA care ranged from 48.2% for major car accident to 89.5% for cold or flu symptoms. Furthermore, students were asked an all-encompassing question regarding their willingness to receive care from a PA. Initially, 52.7% of students were willing to receive most care from a PA. After the intervention, 74.8% of students were willing to receive most care from a PA. This was a statistically significant increase at *p*<0.001 (Table 7).

**Table 6:** Comparison of Initial and Final Perceptions of Undergraduate Students by Medical Problem *Comparison significant at p<0.05.

Medical Problem	Yes Initial (%)	Yes Final (%)	*p*-value
Deep wound	39.1	69.8	<0.001*
Cold or flu symptoms	88.6	89.5	0.11
Chest pain	54.6	76.2	<0.001*
Trouble breathing	53.5	79.2	<0.001*
Abdominal pain	56.5	78.7	<0.001*
Trouble urinating	57.3	77.3	<0.001*
Blood in your stool	44.6	72.9	<0.001*
Sprained ankle	70.6	84.2	<0.001*
Rash	79.5	86.1	<0.001*
Headache	86.7	88.4	0.046*
Minor car accident	54.6	81.2	<0.001*
Major car accident	21.6	48.2	<0.001*
Depression	42.1	68.1	<0.001*
Diabetes	39.3	71.2	<0.001*
High blood pressure	59.0	78.4	<0.001*
Sexually transmitted infection	44.3	77.3	<0.001*

**Table 7:** Initial and Final Willingness to Receive Most, Some, or None of Care from a PA * Comparison significant at p<0.05 when comparing “most” to “some + none” pre-intervention and post-intervention.

Level of Care	Yes Initial (%)	Yes Final (%)	*p*-value
Most	52.7	74.8	<0.001*
Some	44.4	22.5	
None	2.8	2.2	

Finally, after delivery of the educational intervention, students were asked their opinion regarding the most appropriate name for the profession. A physician assistant (keeping the name the same) garnered the most votes at 33.9%, followed by medical practitioner at 30.6%, and three other titles at less than 15% each. It is notable that 66.1% of students preferred a title that was different from the current professional title of physician assistant (Table 8).

**Table 8:** Surveyed Students' Selection for Most Appropriate Profession Name

Title	Percent
Physician assistant	33.9
Physician associate	13.8
Medical practitioner	30.6
Assistant physician	10.8
Advanced practice clinician	10.8

## Discussion

This was a cross-sectional study examining knowledge and perceptions of the PA profession among undergraduate college students. The results demonstrate that, although knowledge of some areas of PA practice is substantial, there are several gaps in knowledge regarding the profession, and there are several subgroups of the college student population who are more knowledgeable than others. Additionally, the results demonstrate a fair perception of PAs at baseline, and a much-improved perception of PAs after a short informational intervention regarding the profession was provided to students.

In general, older students (either defined by age 22-23 or by the class standing of junior or senior) had significantly more knowledge of the profession than younger students. This could potentially be explained by these students likely having had more contact with the healthcare system. Additionally, it may be that older students begin to more seriously consider career options, and those students with healthcare career aspirations may have read about the profession in exploring their potential career options. Females also had more knowledge regarding the profession than males. This may be explained because females may have had more contact with the healthcare system through obstetrics and gynecological appointments for which there is no male counterpart. Furthermore, the PA profession tends to attract more females than males, as approximately 66% of enrolled students are females [12]. It may be that female students are more knowledgeable because they are more interested in the profession.

Additionally, students who planned for a career in health care or who were majoring in a health or human services field had significantly more knowledge of PAs than those who did not plan a career in that field or pursue an education in a health-related field. This is likely due to students having researched the different career options available to them in the healthcare arena or having worked or volunteered in some capacity in healthcare. Interestingly, students who reported receiving care from a PA did not have increased knowledge of the profession compared to those who did not report receiving PA care. This may be due to students confusing physician assistant with another healthcare worker, such as a medical assistant. However, it may also in part be due to PAs not adequately educating patients regarding the role and responsibilities of PAs. Further research is needed to evaluate how effectively PAs educate patients regarding their role, as this represents a potential area where improvements can be made so that college students become more knowledgeable regarding the profession.

In examining the responses to knowledge questions regarding PA abilities to care for patients, several trends emerge. Firstly, most students agreed that PAs could perform tasks typically delegated to a medical assistant, including taking vitals and checking height and weight. However, as the tasks became more complex or required more knowledge, students were less likely to be knowledgeable that PAs were able to perform these tasks. Less than 40% of participants were knowledgeable that PAs could prescribe medications, diagnose illness, treat patients in the emergency room, staple closed a head wound, suture a patient to close an operation, or perform a skin biopsy, among others. These, of course, are tasks that PAs perform every day, depending on the chosen specialty, and highlight the lack of understanding among students regarding PA education and training. This also highlights the importance of PAs educating this population when encountered in a clinical setting, as it is an opportunity to improve knowledge and provide patients with enhanced comfort regarding PA provider skills and expertise. Furthermore, this provides evidence for the need for further marketing of the PA profession to this population.

Evaluating the initial perception of the profession among different subgroups of college students also yielded interesting results. Older students (either defined by age 22-23 or by the class standing of junior or senior) had a better perception of the profession than younger students. This could be because of greater contact with the healthcare field and more opportunity for positive experiences with PAs. This also may reflect that older students have done more research into potential careers, and may have come across one of several recent media articles lauding PAs as a noteworthy profession for students to pursue. Also, students who planned to pursue a career in the healthcare field had a better perception of PAs, likely reflecting their research into potential career options and positive media portrayal of the profession. Finally, students who received care from a PA or who were satisfied with their care from a PA had better perceptions of PAs. This is expected given that most students who received care from a PA were satisfied with that care, likely leading to a positive perception of the profession.

After providing a very short educational intervention, PA perception significantly improved. Scores improved in each subset of the population, and there was a statistically significant improvement in positive responses to every perception question with the exception of one question regarding a PA's ability to care for someone with cold and flu symptoms, which had a very high perception at baseline. This reveals that a short educational intervention can make a large difference in perception of the profession among students, and further underscores the importance of PAs taking the time to educate their patients. Furthermore, after this intervention, students were asked a question regarding the most appropriate name for the PA profession going forward. Though keeping the current name of physician assistant received the most votes, approximately two-thirds of students made other choices and a nearly equal number of students preferred the title of medical practitioner. This may provide an area of future research both among students and the general population as to what name would best fit the profession and be the least confusing to them going forward.

There are several limitations that should be considered in interpreting the above results. Due to the cross-sectional study design, a causality of relationships between the independent and dependent variables cannot be inferred from any significant findings. Data for this study were self-reported and relied upon students’ honest answering of the provided questions. As this study only comprises participants from one university, results of the research are likely only generalizable to students at other universities with similar student populations. A study involving multiple universities from with different student demographics is needed and may be more generalizable to the population of college students as a whole. Additionally, there are limitations as a result of the instrument. Though appropriate measures were taken to ensure that the instrument is a valid measure of knowledge and perceptions, the instrument had not been previously validated or studied, and this must be taken into consideration when interpreting the research findings. Lastly, although statistical methodology controlled for some confounding variables during data analyses, data for all possible confounding variables was not available for use in this study as not all potential confounders were known, nor could data on all of them feasibly be collected.

This study results in several important recommendations. Because there are significant gaps in knowledge of the PA profession among college students, educational interventions should be developed to target this population. This may include on-campus courses exploring healthcare-related fields or even interventions to be implemented by healthcare providers, particularly PAs, when taking care of this population. Other options to improve knowledge include marketing campaigns geared towards this population or the public at large from hospitals or PA organizations. 

These interventions will likely also serve to improve perceptions of PAs, given the impact on perceptions that a small educational intervention had on college students in this study. Improving perception is important to enable PAs to practice as autonomous providers within the healthcare field, to continue to encourage bright minds to seek careers in the field, to further the growth of the profession, and to enable meaningful legislative change to take place regarding PA scope of practice. Future research is needed to determine which of these potential options, or others, may be the most beneficial in reaching the target audience and having the intended impact. Future research is also needed, both on the knowledge and perceptions of the entire United States population, particularly as the profession becomes more well-known and popular so that such interventions can be appropriately created to optimize their impacts, fill the gaps in knowledge, and maximally improve perceptions. Additionally, such research will allow measurement of change in knowledge and perception over time in response to these interventions.

Finally, future research is needed into what the best title for the profession would be going forward. Given that the term “assistant” may not be an adequate representation of their role and capabilities, and also may be misleading to the public, this research would help to bring clarity to this controversy. Furthermore, given that two-thirds of students preferred a name other than physician assistant for the profession, there is at least now some evidence upon which to base this further exploration. 

## Conclusions

In conclusion, several demographic variables are associated with the knowledge and perception of PAs among undergraduate college students, and there is much room to improve that knowledge and perception. Through a brief educational intervention, significant improvements in perception were seen. This study identifies areas of needed improvement in knowledge and perception of PAs and also provides the impetus for educational and marketing-based interventions to improve the knowledge and perception of the PA profession in this population.
